# Deoxyinosine repair in nuclear extracts of human cells

**DOI:** 10.1186/s13578-015-0044-8

**Published:** 2015-09-08

**Authors:** Chia-Chia Lee, Ya-Chien Yang, Steven D. Goodman, Shi Chen, Teng-Yung Huang, Wern-Cherng Cheng, Liang-In Lin, Woei-horng Fang

**Affiliations:** Department of Clinical Laboratory Sciences and Medical Biotechnology, College of Medicine, National Taiwan University, #7, Chung-Shan South Road, Taipei, 10002 Taiwan ROC; Department of Laboratory Medicine, National Taiwan University Hospital, Taipei, 10002 Taiwan ROC; Center for Microbial Pathogenesis, Nationwide Children’s Hospital, Columbus, OH USA

**Keywords:** Deoxyinosine repair, Mismatch repair, Human cell extracts, In vitro assay, DNA repair deficiency

## Abstract

**Background:**

Deamination of adenine can occur spontaneously under physiological conditions generating the highly mutagenic lesion, hypoxanthine. This process is enhanced by ROS from exposure of DNA to ionizing radiation, UV light, nitrous acid, or heat. Hypoxanthine in DNA can pair with cytosine which results in A:T to G:C transition mutations after DNA replication. In *Escherichia coli*, deoxyinosine (hypoxanthine deoxyribonucleotide, dI) is removed through an alternative excision repair pathway initiated by endonuclease V. However, the correction of dI in mammalian cells appears more complex and was not fully understood.

**Results:**

All four possible dI-containing heteroduplex DNAs, including A-I, C-I, G-I, and T-I were introduced to repair reactions containing extracts from human cells. The repair reaction requires magnesium, dNTPs, and ATP as cofactors. We found G-I was the best substrate followed by T-I, A-I and C-I, respectively. Moreover, judging from the repair requirements and sensitivity to specific polymerase inhibitors, there were overlapping repair activities in processing of dI in DNA. Indeed, a hereditable non-polyposis colorectal cancer cell line (HCT116) demonstrated lower dI repair activity that was partially attributed to lack of mismatch repair.

**Conclusions:**

A plasmid-based convenient and non-radioisotopic method was created to study dI repair in human cells. Mutagenic dI lesions processed in vitro can be scored by restriction enzyme cleavage to evaluate the repair. The repair assay described in this study provides a good platform for further investigation of human repair pathways involved in dI processing and their biological significance in mutation prevention.

## Background

Deoxyinosine (hypoxanthine deoxyribonucleotide, dI) in DNA can arise from spontaneous deamination of deoxyadenosine residue, and is also induced by ROS produced from normal aerobic respiration. In addition, exposure of DNA to ionizing radiation, UV light, nitrous acid, or heat can promote the formation of dI [1, 2]. Alternatively, dI can be introduced by misincorporation of dITP in the nucleotide pool during replication [3, 4]. Deoxyinosine derived from deamination of deoxyadenosine in DNA is potentially mutagenic since it prefers to pair with dCTP during replication, yielding A:T to G:C transition mutations at sites of adenine deamination [5].

In mammalian cells, base excision repair (BER) was thought to be the major pathway for dI repair. The excision of base damage is initiated by a specific DNA glycosylase: Hypoxanthine is bound and excised efficiently by human *N*-methylpurine-DNA glycosylase (MPG, also known as AAG, ANPG, APNG, or MDG) [6]. From radionucleotide incorporation fine mapping, the resulting apurinic/apyrimidinic (AP) sites are further processed by both the short patch pathway (1-nucleotide gap filling) with DNA polymerase (Pol) β and the long patch pathway (2-6 nucleotide resynthesis) with Pol δ and PCNA [7].

In *Escherichia coli*, early studies indicated that the DNA glycosylase encoded by *alkA* gene could recognize and release hypoxanthine residues from DNA [1]. However, subsequent in vivo and in vitro studies showed that DNA glycosylase initiated BER is not the major pathway to process dI in *E. coli* [8, 9]. The repair pathway initiated by endonuclease V (EndoV, encoded by *nfi* gene [10]) was shown to be the major pathway for dI processing both in vivo and in vitro [8, 9, 11]. A mutagenesis assay also showed that under HNO_2_ treatment, which will promote hypoxanthine formation, that a *nfi* mutant demonstrated over a 200-fold increase in mutation frequency, while the *alkA* mutant did not significantly increase the mutation frequency under the same experimental conditions [12].

Endonuclease V (EndoV) in *E. coli* is active upon DNA exposed to UV light, OsO4, acids, or X-rays [10]. This enzyme was later characterized as 3′-deoxyinosine endonuclease that incises the DNA at the second phosphodiester bond 3′ to the dI lesion, leaving 3′-OH and 5′-P termini [13]. *Nfi* homologues from *Thermotoga maritima* possess 3′-exonuclease activity that might be used for removal of damaged bases [14], but similar exonuclease activities were not found in EndoV from *E. coli* and mammalian cells. Therefore, additional enzymes are required to excise the dI lesions in the EndoV-mediated repair process. In our previous study, we found DNA pol I played dual roles in both repair synthesis and using its 3′-5′ proofreading exonuclease to remove EndoV incised dI lesion [9, 11].

A mammalian homologue of *E. coli nfi* gene was identified and characterized [15]. The mouse EndoV seems to be active only on dI, while bacterial EndoV exhibits broad substrate spectrum. Furthermore, expression of mouse EndoV in an *alkA*, and *nfi* double mutant *E. coli* strain significantly suppresses the spontaneous mutagenesis frequency, which suggested that this eukaryotic EndoV initiates an alternative excision repair pathway for dI correction [15]. A biochemical analysis of purified human EndoV showed it favored dI-containing DNA but with only a minor preference on deoxyxanthosine-containing DNA [16]. Expression of hEndoV in *E. coli* cells deficient in *nfi*, *mug* and *ung* genes caused 3-fold reduction in mutation frequency [16]. However, recent reports demonstrated efficient cleavage of inosine-containing RNA by human EndoV [17] suggesting that hEndoV may involve in RNA editing [18]. Therefore, the full involvement of hEndoV in dI repair in human cells is still unknown.

The major function of mismatch repair (MMR) is its role in correction of nucleotide base misincorporation during replication [19–21], which requires that repair be directed to a newly synthesized DNA strand. A strand-specific nick or gap is sufficient to direct MMR in extracts of mammalian cell extracts, and an obvious possibility is that DNA termini that occur naturally at the replication fork serve as the strand signals that direct the reaction in the eukaryotic cell [19]. Several purified systems have been reconstituted using near homogeneous human proteins and support 3′- and/or 5′-directed mismatch-provoked excision or repair. According to the current model, the mismatch recognition activities MutS-α (MSH2·MSH6 heterodimer) or MutS-β (MSH2·MSH3 heterodimer), MutL-α (MLH1·PMS2 heterodimer), RPA (replication protein A), the 5′–3′ double-strand hydrolytic activity exonuclease 1 (Exo1) [19], the replication clamp proliferating cell nuclear antigen (PCNA) and the clamp loader replication factor C (RFC) yields a system that supports mismatch-provoked excision directed by a 3′ or 5′ strand break, and where hydrolysis is attenuated upon mismatch removal [19].

The roles and sequence events utilizing each of the aforementioned proteins have at least been partially elucidated. RPA plays a primary role in terminating excision by MutS-α-activated Exo1; additionally high mobility group protein B1 (HMGB1) was found to substitute for RPA [19]. MutL-α is a latent endonuclease that is activated in a manner that depends on a mismatch, MutS-α, RFC, PCNA, ATP and a strand break. While the function of RFC in MutL-α activation is apparently restricted to clamp loading, the PCNA loading orientation determines the strand direction of MutL-α incision, targeting endonuclease action to the heteroduplex strand that contains a preexisting break [22, 23]. Incision in this manner introduces additional breaks, providing a 5′ nick that serves as a loading site for MutS-α-activated Exo1, which removes the mismatch. Upon completion of the mismatch excision process [24], DNA pol δ synthesizes DNA in the place of the excised sequence and DNA ligase I then joining any nicks in the DNA sequence [21]. While this human mismatch repair pathway may recognize base analogs and damaged bases [25] our previous attempts to show in vitro processing of a dI containing heteroduplexes by bacterial MutHLS mismatch repair pathway was insignificant [9, 11]. However the possibility of overlapping repair activities for dI process in human mismatch repair was not tested.

In this study a convenient and non-radioisotopic method was used to study dI repair in mammalian cells. Specifically, we took advantage of a functional assay that uses DNA substrates containing a dI residing in the recognition site or cleavage site of restriction enzymes. Substrates processed by human cell-free extracts can be scored by restriction enzyme cleavage to evaluate the repair of dI. We found that all four dI lesions of A-I, C-I, G-I and T-I can be processed by human extracts with different efficiencies. We also performed a comparative examination of repair requirements of different dI containing substrates in vitro and the results indicated that there were overlapping activities of several repair pathways in processing of dI in DNA.

## Results

### Deoxyinosine-containing substrates are efficiently processed in Hela cell extracts

**Fig. 1:**
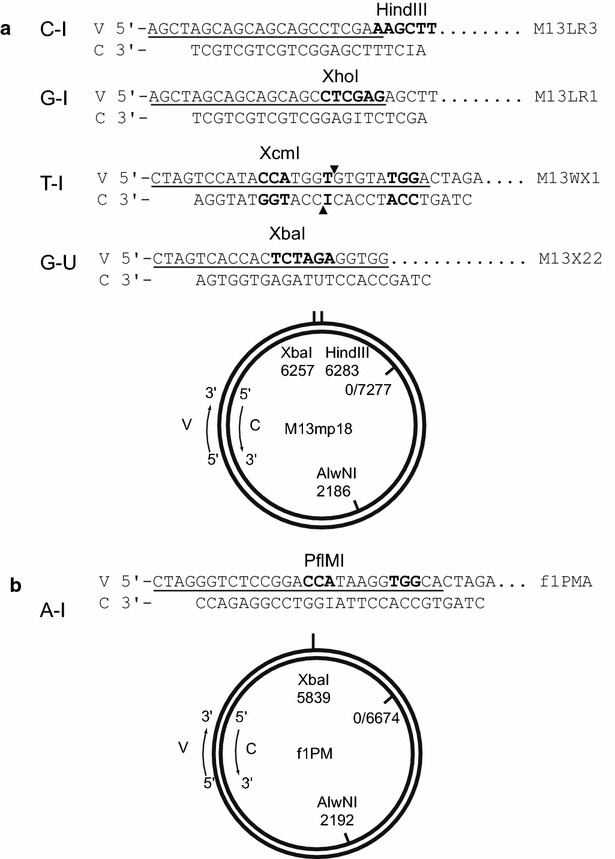
Map of M13mp18 and f1PM based heteroduplex substrates. **a** The map of bacteriophage M13mp18 replicative form (RF) DNA shows restriction enzyme sites relevant to this study with derivatives M13LR1 and M13LR3 containing 22-bp insertions at the unique HindIII restriction site, and phage M13WX1 and M13X22 containing 26-bp and 22-bp insertions at *Xba*I site respectively. **b** The map of bacteriophage f1PM RF DNA with its derivative f1PMA with a 27-bp insertion at *Xba*I. ‘V’, phage viral strand. ‘C’, phage complementary strand. Underlines beneath each viral strand are the original insertion sequences. The C-strand from parental phage RF DNA was paired with viral strand of its insertion derivative to produce gapped duplex DNA, and the gap was sealed with dI or deoxyuridine containing synthetic oligodeoxyribonucleotide. A-I, C-I, G-I, T-I, and G-U are the resulting substrates and DNA sequence shown on each C-strand of the the synthetic linker used. In the presence of dI, the substrates were refractory to the restriction endonuclease scoring. After the repair, DNA products become sensitive to restriction endonuclease cleavage. The recognition sequence of corresponding restriction endonuclease markers for repair products are shown in bold on V-strands

Previously, to evaluate EndoV repair system in *E. coli*, we constructed a set of dI-containing substrates A-I, G-I and T-I heteroduplexes [9, 11]. In these substrates, a dI resided in a disrupted restriction endonuclease recognition or cleavage site (Fig. 1). We employed a restriction endonuclease assay to score for the repair of dI. In the presence of dI lesion, heteroduplex DNA is refractory to restriction endonuclease cleavage. After in vitro repair the specific restriction endonuclease recognition sequence was restored and repair level can be scored by the extent of restriction digestion [9, 11]. In this study, we added newly constructed C-I substrate (Fig. 1) to this assay platform and extended our study to determine the dI repair efficiency in human nuclear extracts.

**Fig. 2:**
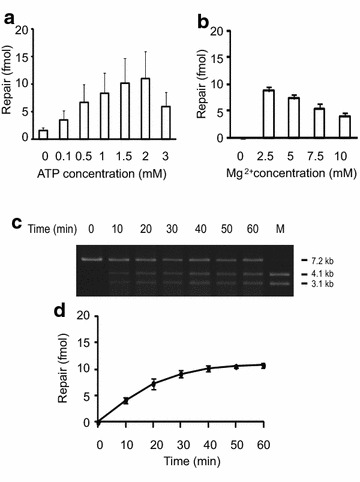
ATP, Mg^++^ titrations and time course of G-I repair in human nuclear extracts. Each reaction contained 90 μg HeLa extracts and 21 fmol G-I substrate. **a** Repair reactions with HeLa extracts were determined as described in Methods except that where indicated ATP was added. **b** Repair reaction with HeLa extracts were performed as described in Methods except where indicated Mg^2+^ was added. **c** Reactions were performed under standard conditions and scaled; 10-μl samples were removed as indicated. DNA products were digested with AlwNI and XhoI and then subjected to agarose gel electrophoresis to score for the repair of dI lesion. The bar pointing to the 7.2-kb fragment represents unrepaired substrate linearized with AlwNI; size marker (M) of 4.1 and 3.1-kb fragments are indicative of repaired products that were generated by treatment of M13LR1 with AlwNI and XhoI. **d** Quantitative analysis of the time course reaction, the error bars represent S.D. from three determinations

We first tested G-I repair in nuclear extracts from a HeLa cell line. In a preliminary trial, reaction conditions for both *E. coli* dI repair [9, 11] and human MMR [26] were tested. To our surprise, the G-I substrate was only marginally repaired by human nuclear extracts in reaction buffer for *E. coli* dI repair (Fig. 2a, 0 mM entry), but in human MMR reaction buffer, it was efficiently corrected (Fig. 2a, 1 mM entry). The major difference in these two reaction buffers was human MMR buffer contained 1 mM ATP while *E. coli* dI repair buffer contained no ATP. We therefore did an ATP titration experiment as shown in Fig. 2a. We found the presence of ATP could stimulate human dI repair. The highest level of correction occurred at a concentration of 2 mM ATP; higher ATP concentrations (>2 mM) caused extensive degradation of DNA and therefore inhibited the repair (Fig. 2a). We also performed a Mg^2+^ titration experiment as shown in Fig. 2b. The best repair level was at 2.5 mM, while higher Mg^2+^ concentrations were inhibitory.

A time course showing the amount of repaired products of a G-I heteroduplex when incubated with HeLa extracts is shown in Fig. 2c, d. Digestion of the unprocessed heteroduplex G-I with AlwNI and XhoI, whose recognition sequence for XhoI is blocked by the presence of dI lesion, will yield a 7.2-kb linear fragment only (Fig. 2c, 0 min). However, digestion of DNA with both enzymes in which the XhoI recognition sequence has been restored by repair will yield 4.1- and 3.1-kb fragments (Fig. 2c, 10–60 min). The repair efficiency can be determined by measurement of band intensity (Fig. 2d).

Although higher ATP concentrations (1.5–2 mM in Fig. 2a) and lower Mg^2+^ (2.5 mM in Fig. 1b) showed better dI repair activity, we decided using conventional human MMR reaction buffer containing 1 mM ATP and 5 mM Mg^2+^ for standard dI repair assay in order to compare dI correction versus human MMR activities described in the subsequent section. As shown in Table 1, in human MMR buffer, A-I, C-I, G-I and T-I can be repaired with different efficiencies. The G-I heteroduplex was most efficiently corrected, and followed by T-I, A-I, and C-I substrates. The levels of repair were about 25–60 % of our previous observed bacterial reactions [9, 11].

**Table 1:** Repair requirement of dI-containing heteroduplex in extracts from MMR-proficient and MMR-deficient mammalian cells

Reaction condition	Repair levels (fmol)							
	HeLaS3				HCT116			
	G-I	T-I	A-I	C-I	G-I	T-I	A-I	C-I
Standard	7.6 ± 1.3	3.8 ± 0.8	3.0 ± 0.6	2.4 ± 0.2	3.2 ± 0.5	2.3 ± 0.6	1.1 ± 0.3	0.9 ± 0.09
Mg^2+^ (-)	–***	–***	–***	–***	–***	–***	–***	–***
dNTP (-)	1.7 ± 0.1***	1.2 ± 0.2***	0.6 ± 0.1***	1.0 ± 0.1**	1.2 ± 0.4**	1.1 ± 0.8	–***	0.7 ± 0.09
ATP (-)	4.7 ± 0.2***	2.8 ± 1.0	2.0 ± 0.3^**^	1.1 ± 0.1**	2.2 ± 0.6	1.5 ± 0.2**	0.8 ± 0.02	0.7 ± 0.07*
ATPγS	4.3 ± 0.01***	1.0 ± 0.4***	0.7 ± 0.1***	1.1 ± 0.1**	2.9 ± 0.6*	0.3 ± 0.4***	1.0 ± 0.2	0.8 ± 0.02

Repair efficiency was determined as described in “Methods”. Twenty-one fmol of dI-containing DNA was incubated with 90 μg of HeLaS3 or HCT116 cell-free extracts at 37 °C for 30 min. Repair level lower than 0.3 fmol was regarded as background (–). Each data corresponds to the average and standard deviation from at least three independent measurements. Statistical analysis: * p < 0.05, ** p < 0.001, *** p < 0.001 versus Standard reaction (Student’s t-test)

### Reaction requirements of dI repair in human extracts

In human dI repair, we adopted reaction conditions of in vitro human mismatch repair for comparison. It is known that in vitro human mismatch repair requires the addition of MgCl_2_, ATP and the four dNTPs [27]. In order to understand if all of these components are essential for dI repair, we systematically omitted the exogenous cofactors in separate reactions. As shown in Table 1, in the absence of Mg^2+^, the repair efficiency of all four dI substrates were reduced below the level of detection in HeLa extracts, which is consistent with that Mg^2+^ being an essential cofactor for most DNA repair enzymes [7, 15, 27]. In the absence of exogenous dNTPs, the repair levels dropped to less than one-third of the standard reactions. The cell-free extracts prepared for this study may contain trace amount of dNTPs [26] and may contribute to these residual repair levels.

In the absence of exogenous ATP, the relative repair levels of each substrate showed variable degrees of decreasing activity when compared to standard ones (Table 1, HeLa entries). This is in contrast to our previous studies in *E. coli* where ATP was not required in bacterial dI repair [9, 11]. This effect might be caused by the fact that the human system employs multiple ATP-dependent repair proteins, for example, human uses ATP-dependent DNA ligase while *E. coli* uses NAD^+^-dependent ligase, respectively. The low level repair in the absence of exogenous ATP might be due to trace amounts of ATP in the cell-free extracts we prepared. Thus the increase in repair by addition of exogenous ATP might be due to insufficient ATP levels for some of the repair proteins. To clarify the role of ATP in the reaction, ATPγS, an ATP analog that is resistant to hydrolysis, was included in the reactions. In the G-I repair reaction, omitting ATP and addition of ATPγS showed a similar limiting of repair (Table 1). However, the repair efficiency of T-I substrates with HeLa extracts showed an extensive reduction when exogenous ATP was replaced by ATPγS in the repair reactions, with the repair levels in ATPγS reactions being even lower than the reactions omitting ATP. Therefore, the presence of an ATP cofactor and its hydrolysis were likely both essential for human T-I lesion repair. To determine if the ATP dependence was nucleotide triphosphate specific, we also tested the effects of exogenous GTP addition from 0.5 to 2 mM to G-I repair reactions; no obvious change of repair levels with GTP addition was observed compared to conditions without exogenous ATP (data not shown).

### Both aphidicolin- and lithocholic acid-sensitive DNA polymerases were involved in correcting dI in mammalian cells

**Fig. 3:**
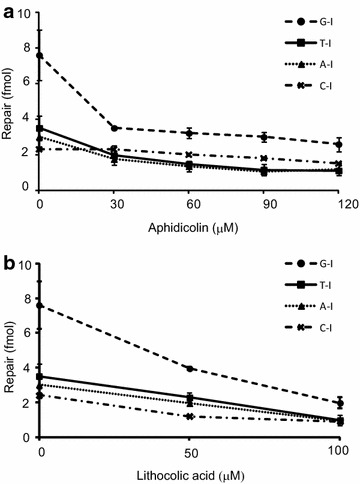
Inhibitory effects of aphidicolin and lithocholic acid on dI correction in mammalian cell-free extracts. Heteroduplexes G-I, T-I, C-I and A-I (21 fmol) were incubated with 90 μg HeLa extracts containing indicated DNA polymerases inhibitors for 30 min at 37 °C. Reactions were analyzed by gel electrophoresis after restriction endonuclease digestion with AlwNI and the scoring enzymes. **a** Addition of aphidicolin. **b** Addition of Lithocolic acid. Each data corresponds to the average and standard deviation (*error bars*) from at least three independent reactions

Several classes of DNA polymerases have been identified in different DNA repair pathways. For example, pol δ is involved in MMR [28, 29], nucleotide excision repair [30, 31] and long-patched BER [32] pathways. In classic mammalian short-patched BER pathway, pol β is responsible for the repair DNA synthesis [33, 34]. In order to evaluate the involvement of DNA polymerases in repairing dI, an inhibitor targeting pol α, δ and ε was added into the repair reactions. Aphidicolin has been found to block the repair DNA synthesis in MMR system [26, 27]. The repair of C-I was not sensitive to aphidicolin inhibition (Fig. 3a). The repair of G-I, T-I, and A-I were significantly reduced by addition of aphidicolin at the concentration of 30 μM in HeLa extract-containing repair reactions (Fig. 3a). However, as the concentration of aphidicolin increased to above 90 μM, at which pol δ dependent MMR should be abolished [26, 27], relatively high levels of residual dI repair remained. This observation suggested both aphidicolin-sensitive and aphidicolin-resistant DNA polymerases participating dI lesions repair, in addition to possible overlapping repair mechanisms for dI repair in human cells.

Deoxyinosine was thought to be repaired by base excision repair in mammalian cells [35, 36]. In classic mammalian short-patched BER pathway, pol β is responsible for the repair DNA synthesis. A bile acid derivative lithocholic acid (LCA) can bind to pol β and disrupts its AP lyase ability to block the DNA replication functions, with the *Ki* value of 10 μM [37]. We therefore introduced this DNA polymerase β inhibitor into dI repair reactions in human extracts. As shown in Fig. 3B, the repair levels of dI heteroduplex DNA were impaired with lithocholic acid treatments with a dose dependency. This observation suggested that pol β and possibly short-patched BER are involved in repairing dI lesions in mammalian cells. At the concentration of LCA at 50 μM, at which pol β activity should be near background level [37], relatively high levels of residual dI repair remained (more than 50 % of activity relative to standard reactions). Since pol δ and ε were not affected by 50 μM LCA [37], it’s possible these LCA-resistant DNA polymerases participate in repair of dI lesions.

### A hereditary non-polyposis colorectal cancer cell line showed lower repair activity toward certain dI lesions

It is known that mismatch repair activity not only can repair mismatches, but also may recognize base analogs as well. The A-I, C-I, G-I and T-I substrates we prepared share a great deal of similarity to mismatches. It is also known that ATP is required in the mismatch repair pathway and our exogenous ATP and ATPγS experimental data were strongly suggested possible involvement of mismatch repair in human dI lesion repair. In order to clarify this issue, we employed extracts from a mismatch repair deficient HCT116 cells in our dI repair assay.

To ensure the quality of both HeLa and HCT116 extracts were comparable, a G-U substrate (Fig. 1) was prepared to evaluate their BER activity. Using the repair assay conditions described above, both cell extracts could actively repair G-U substrate. In a reaction of 21 fmol G-U substrate, repair levels from HeLa was 17.9 ± 0.2 fmol and from HCT116 was 17.4 ± 0.3 respectively.

However, we found the repair levels of HCT116 decreased about 60 % in G-I, 35 % in T-I, 66 % in A-I, and 38 % in C-I substrates when compared to repair levels derived from HeLa cells (Table 1). We also determined the repair requirements for HCT116 extracts (Table 1). In the absence of exogenous dNTPs, the repair levels dropped to near background for A-I to less than half of the standard reactions for G-I and T-I (Table 1). In the absence of exogenous ATP, the repair levels of HCT116 for each substrate also showed variable degrees of decreasing repair when compared to standard ones (Table 1, HCT116 entries). It is very interesting to note that the repair efficiency of T-I showed an extensive reduction in both HeLa and HCT116 extracts when exogenous ATP was replaced by ATPγS in the repair reactions. This implies that the T-I specific repair mechanism exists in human cell relies on ATP hydrolysis.

**Fig. 4:**
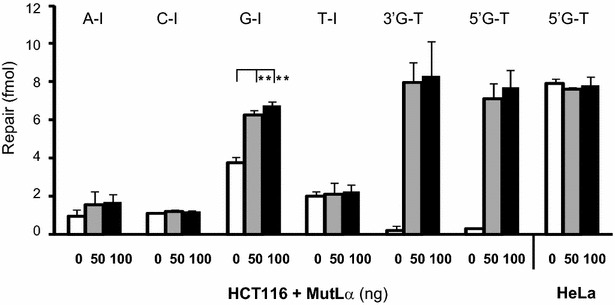
MuL-α complementation assay for Hct116 extracts. Heteroduplex A-I, C-I, G-I, T-I, 3′G-T and 5′G-T (21 fmol) as indicated were incubated with 90 μg Hct116 extracts in the absence (*white bar*) or presence of 50 ng (*gray bar*) or 100 ng (*black bar*) of human MutL-α recombinant protein. Repair of 5′G-T with HeLa was included as a control. Each *data-bar* corresponds to the average and standard deviation (*error bars*) from at least three independent reactions. **p < 0.01 versus addition of exogenous MutL-α (Student’s *t* test)

Since HCT116 lacks functional MutL-α for mismatch repair, the observation described above prompted us to test the involvement of MutL-α in human dI repair. As shown in Fig. 4, supplemented purified human MutL-α to MMR proficient HeLa extracts showed no significant change in 5′G-T repair levels (Fig. 4, HeLa entries). However, supplementing of MutL-α to HCT116 extracts was able to restore its MMR activity for both 3′-G-T and 5′-G-T heteroduplexes. Addition of MutL-α to HCT116 extracts also increased the G-I repair level comparably to that of HeLa extract containing reactions (Fig. 4 and G-I entry in Table 1). However, the processing of A-I, C-I and T-I showed no significant change in the presence or absence of MutL-α (Fig. 4). This observation suggested human MMR enzymes can recognize and process G-I but not A-I, C-I and T-I lesions.

## Discussion

Deamination of purine bases can occur spontaneously. The conversion rate of adenine to hypoxanthine in DNA under physiological condition is about 2 % of the rate of the conversion of cytosine to uracil [2], which in turn is about 2–5 times per human cell per day [38]. The resulting I-T mismatch in DNA is potentially mutagenic since a dI in the DNA template prefers to pair with dCTP during replication, yielding A:T to G:C transition mutations at sites of adenine deamination [5]. Therefore dI in DNA should be removed by living organisms to maintain genome integrity.

Previously we developed an in vitro assay for bacterial repair and found that different dI-containing substrates were predominately repaired by the EndoV repair pathway [11]. In this study, we took advantage of our previously described bacterial dI in vitro repair assay to determine repair capabilities in human cell-free extracts for all four dI-containing A-I, C-I, G-I and T-I substrates. Similar to *E. coli*, we found that human cell extracts can also efficiently process dI lesions. However, some significant differences do exist between human and bacterial reactions. In the reactions withholding specific exogenous cofactors, we found that high concentrations of exogenous ATP was required for the human reaction (Table 1) but not in the bacterial reaction [11], This observation might be attributed to some of mammalian DNA processing proteins requirement for ATP for their enzymatic reactions. Mammalian DNA ligases, PCNA loader (RFC), MutS-α, MutL-α are among the likely ATP utilizing candidates involved in the reaction.

As to substrates specificity, under similar reaction conditions, we found G-I was the best substrate for human cell extracts followed by T-I, A-I and C-I. This hierarchy of repair specificity in human cells is different from what we previously reported in the bacterial system i.e. T-I is a better substrate than A-I and G-I [11]. In *E. coli*, both in vivo and in vitro studies suggested that EndoV pathway is the major system for processing dI lesions. All the mutagenic dI-containing DNA of A-I, G-I and T-I were repaired with similar biochemical process in vitro [11] and with the same genetic requirements for in vivo correction [8]. However, correction of dI in human extracts appears to be more complex. We suspect there are multiple overlapping dI repair activities judging from different repair responses for G-I, T-I, C-I and A-I with specific inhibitors.

It is not uncommon for repair proteins or pathways to demonstrate overlapping specificity toward the same DNA errors or lesions. For example, to deal with cytosine deamination, mammalian cells utilize four nuclear uracil DNA glycosylases (UDGs), namely, UNG2, SMUG1, TDG and MBD4 [39]. Likewise, BER was thought to be the major pathway for dI repair in mammalian cells. According to the model, hypoxanthine (Hx) would be bound and excised relatively efficiently by human *N*-methylpurine-DNA glycosylase (MPG, also known as AAG, ANPG, APNG, and MDG) [6]. The human MPG has a broad substrate specificity, excising a structurally diverse group of modified purines from DNA [40]. Single-turnover kinetics of excision of Hx paired with T showed that excision of Hx was very fast relative to any other damaged purines. However, the opposing pyrimidine base had a significant effect on the kinetics of excision and DNA binding affinity of Hx; replacing a T with a U opposite Hx dramatically reduced the excision rate [35]. In mammalian cells, pol β is responsible for the dominant 1-nucleotide replacement or so-called short-patch BER pathway. In long-patch BER pathway, DNA Pol δ or ε, FEN1, PCNA and DNA ligase I are required for gap filling and deoxyribosephosphate removal.

An initial effort toward identifying the requirements in our human dI repair assay was to use DNA polymerase inhibitors. Using lithocholic acid or aphidicolin treatments, the repair of G-I, C-I, T-I, and A-I were partially inhibited in reactions containing HeLa extracts (Fig. 3). This observation implies the possible role for both a lithocholic acid sensitive polymerase (i.e. β) and aphidicolin sensitive polymerases (i.e. α, δ or ε) in dI repair. Different repair specificities toward A-I, C-I, G-I, and T-I substrates and involvement of both lithocholic acid sensitive and aphidicolin sensitive DNA polymerases are an indication of possible involvement of both short- and long- patch BER pathway. Our observation is consistent with a previous report that repair of a hypoxanthine residue initiated by the MPG utilizes both short- and long-patch repair synthesis [7].

The A-I, C-I, G-I and T-I mispairs share some similar features to base–base mismatches and cause protrusion of the DNA duplex [41]. However, in *E. coli*, both in vivo and in vitro studies showed the mismatch repair pathway plays little, if any role in processing dI lesions [8, 9, 11]. Using extracts from an MMR-deficient HNPCC cell line with a complementation assay demonstrated that G-I substrate could be partially processed by MMR pathway (Fig. 4). A notable finding from this study is that we found extracts of HCT116 showed lower dI processing capability compared to HeLa cell extracts. HCT116 is defective in mismatch repair due to a lack of human MutL-α activity indicating a possible role of the mismatch repair protein MutL-α in processing dI lesions. However, we found the deficiency was not completely due to mismatch repair since addition of recombinant MutL-α, which can restore HCT116’s MMR activity, failed to significantly increase the repair levels of T-I, C-I and A-I substrates, while significantly improving G-I repair (more than 50 % increase as shown in Fig. 4). A single-strand break is required to initiate repair and to provide strand specificity for human MMR [26]. Structurally similar to mismatches, covalently-closed-circular G-I substrate may be detected by MutS-α which subsequently stimulates MutL-α nicking activity to initiate the repair [22, 23]. Alternatively, an incision created by an EndoV-like activity [42] might be sufficient to direct the repair. The interaction between EndoV and MMR enzymes for G-I processing remains to be determined.

EndoV from *E. coli* has been shown to have similar activity on oligodeoxynucleotides containing T-I, C-I, G-I or A-I pairs [13]. Recently, the human EndoV homologue was identified and characterized [16]. The mammalian EndoV showed lower endonucleolytic activity than *E. coli* homologues towards dI lesions [15, 16]. The activity of mammalian EndoV was most active on G-I followed by T-I>A-I>C-I [16]. In combination with the overlapping repair of G-I by MMR, this might explain our findings that G-I was most efficiently processed followed by T-I, A-I, and C-I (Table 1). Further study is required to clarify this issue.

Variations in DNA repair activities are connected to both individual and population disease susceptibilities [43, 44], Several DNA repair defects have been linked to cancers [43, 44]. Oxidatively damaged DNA and its repair are also positively correlated in colon carcinogenesis [45]. Increased risk of lung cancer is associated with a functionally impaired polymorphic variant of the human DNA glycosylase NEIL2 [46]. Thus, studying DNA repair activities in human cells is an important issue. A very interesting finding from this study is that we found that HNPCC cell line HCT116 showed lowered dI processing capability in part because of its MMR deficiency (for part of G-I repair) and possibly other mechanism(s) for T-I, C-I and A-I repair. Production of dI can be enhanced by exposure of DNA with nitrite ion. Nitrites are commonly used in the food production industry for curing meat. It’s likely that the digestive tract e.g. the colon would be exposed and affected by nitrite when consuming preserved meat items. Patients of putative HNPCC families might be more susceptible to this mutagenic effect. The interrelationship of a MMR deficiency and other dI repair activities in HNPCC tumors needs to be determined. The dI repair assay described in this study provides a good platform for further investigation.

## Conclusions

In this study a plasmid-based convenient and non-radioisotopic method was introduced to study dI repair in human cells. All four possible dI lesions of A-I, C-I, G-I and T-I processed by human cell-free extracts could be scored by restriction enzyme cleavage to evaluate the repair. In addition, repair derived from a MMR deficient cancer cell line was less efficient in dI repair that was partially attributed to lack of MutL-α. The repair assay described in this study provides a good platform for further investigation of human repair pathways involved in dI processing and their biological significance in mutation and cancer prevention.

## Methods

### Materials

Bacteriophage M13LR1 and M13LR3 were derivatives of M13mp18 with a 22-base pair (bp) insertion at HindIII site [47]. M13WX1 and M13X22 were derivatives of M13mp18 with 26 and 22-bp insertion at XbaI site, and phage f1PM-A was a derivative of f1PM with a 27-bp insertion at XbaI site [11] (Fig. 1). *E. coli* DNA ligase, T4 polynucleotide kinase, HindIII-HF^TM^ and other restriction endonucleases were obtained from New England Biolabs. RecBCD nuclease was purchased from EPICENTRE Biotechnologies. Aphidicolin and lithocolic acid were purchased from Sigma and dissolved in DMSO. Recombinant human MutL-α was kindly provided by Dr. Paul Modrich (Duke University).

### Cell culture and preparation of human cell-free nuclear extracts

The human cell line HeLa S3 were grown in 10 % FBS supplemented DMEM/F12 medium (Sigma), and Hct116, a mismatch repair deficient colorectal carcinoma cell line were grown in 10 % FBS supplemented RPMI 1640 medium (Biological Industry) at 37 °C under a 5 % CO_2_ atmosphere.

Human cell-free nuclear extracts was prepared as described [27]. In brief, human cell lines were cultured to a total cell number about 2 × 10^9^ cells. Cells were washed twice with a buffer of 20 mM HEPES (pH7.5), 5 mM KCl, 0.2 M sucrose, 0.5 mM MgCl_2_, 0.1 % PMSF, and 0.5 mM dithiothreitol, and then incubated in a hypotonic solution of 20 mM HEPES (pH7.5), 5 mM KCl, 0.5 mM MgCl_2_, 0.1 % PMSF, and 0.5 mM dithiothreitol. Cells were lysed with a dounce homogenizer, nuclei were collected by centrifugation. Nuclear protein were extracted in 50 mM HEPES (pH7.5), 10 % sucrose, 0.1 % PMSF, 155 mM NaCl, and 0.5 mM dithiothreitol, and then concentrated by ammonium sulfate precipitation (0.42 g/ml). The pellet was dialyzed against 25 mM HEPES (pH7.5), 50 mM KCl, 0.2 % PMSF, and 1 mM dithiothreitol until the conductivity achieved a value equivalent to that of 0.1–0.2 M KCl. The soluble fraction was frozen in small aliquots in liquid nitrogen and stored at −80 °C.

Batch to batch variation of cell-free nuclear extracts for repair activity was quite high (up to 30 % differences for standard reaction). Higher S.D. in some data reflected using average of equal number measurements from different batch of extracts.

### Deoxyinosine-containing heteroduplex DNA substrates

Preparation of heteroduplex DNA substrates was carried out by annealing a 5′-phosphorylated dI-containing oligonucleotides (Fig. 1) to a gap-duplex DNA and then sealed by T4 DNA ligase as described [11]. All the covalently-closed-circular dI containing substrates were purified by CsCl-Ethidium bromide density gradient centrifugation. The key feature of prepared A-I, C-I, G-I and T-I substrates were summarized in Fig. 1. All of the dI-containing substrates were confirmed to be refractory to respective diagnosis restriction endonuclease cleavage prior the repair reaction.

### Repair assays using cell-free extracts

The deoxyinosine repair assay with human extracts was similar to a human mismatch repair assay as described [26]. Briefly, 21 fmol of DNA substrate was incubated with 90 to 105 μg of human nuclear extracts in a 20-μl reaction containing 20 mM Tris–HCl (pH 7.6), 50 μg/ml bovine serum albumin, 5 mM MgCl_2_, 1 mM ATP, and 0.1 mM each of the four dNTPs. Reactions were incubated for 30 min at 37 °C and quenched by adding 40 μl of 40 mM EDTA (pH8.0). DNA was isolated by phenol extraction and ethanol precipitation, and was then digested with AlwNI and the indicated scoring restriction enzymes. The resulting products were separated by agarose gel electrophoresis, and detected by ethidium bromide staining. The gel-images were captured by a gel documentation CCD camera (UVP Ltd.) using Viewfinder 3.0, and band intensities were then measured by NIH Image J 1.45 s software.

A DNA mismatch repair assay using nicked 5′-GT and 3′-GT heteroduplex was as described [48]. In the MutL-α complementation assay, purified MuL-α protein was supplemented to the HCT116 nuclear extracts and the repair assay was as described above.

A G-U substrate (Fig. 1) was prepared to evaluate BER activity of Hela and Hct116 extracts. The G-U repair assay is similar to dI assay described above.

## Abbreviations

dI: deoxyinosine
MMR: mismatch repair
RPA: replication protein A
Exo1: exonuclease 1
PCNA: proliferating cell nuclear antigen
RFC: replication factor C
BER: base excision repair
Pol: DNA polymerase
EndoV: endonuclease V
LCA: lithocholic acid
